# Crystal structure of poly[{μ-*N*,*N*′-bis[(pyridin-4-yl)meth­yl]oxalamide}-μ-oxalato-cobalt(II)]

**DOI:** 10.1107/S1600536814015608

**Published:** 2014-08-01

**Authors:** Hengye Zou, Yanjuan Qi

**Affiliations:** a Department of Chemistry, Changchun Normal University, Changchun 130032, People’s Republic of China

**Keywords:** crystal structure, metal-organic framework, cobalt(II), oxalate anion, hydrogen bonds

## Abstract

In the polymeric title compound, [Co(C_2_O_4_)(C_14_H_14_N_4_O_2_)]_*n*_, the Co^II^ atom is six-coordinated by two N atoms from symmetry-related bis­[(pyridin-4-yl)meth­yl]oxalamide (BPMO) ligands and four O atoms from two centrosymmetric oxalate anions in a distorted octa­hedral coordination geometry. The Co^II^ atoms are linked by the oxalate anions into a chain running parallel to [100]. The chains are linked by the BPMO ligands into a three-dimensional architecture. In addition, N—H⋯O hydrogen bonds stabilize the crystal packing.

## Related literature   

For information on compounds with metal-organic framework structures, see: Kitagawa *et al.* (2004 ▶); Ma *et al.* (2009 ▶); Li *et al.* (2005 ▶); Wang *et al.* (2007 ▶). For related Co^II^ complexes, see: Ma *et al.* (2005 ▶).
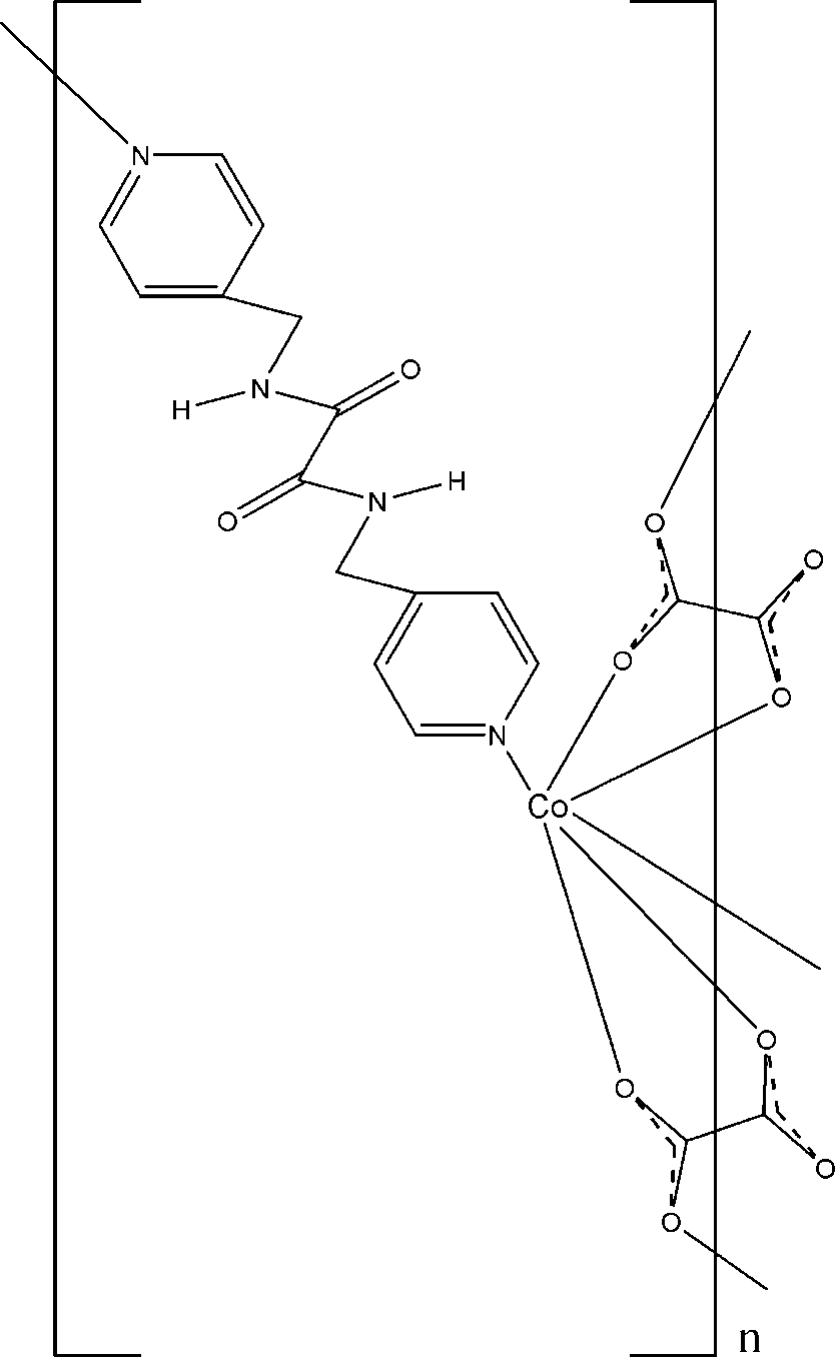


## Experimental   

### Crystal data  

[Co(C_2_O_4_)(C_14_H_14_N_4_O_2_)]
*M* *_r_* = 417.24
Monoclinic, 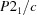

*a* = 8.4143 (12) Å
*b* = 24.421 (4) Å
*c* = 9.2884 (14) Å
β = 113.322 (2)°
*V* = 1752.7 (4) Å^3^
*Z* = 4
Mo *K*α radiation
μ = 1.02 mm^−1^
*T* = 293 K
0.43 × 0.25 × 0.25 mm

### Data collection  

Bruker SMART APEXII CCD diffractometer
Absorption correction: multi-scan (*SADABS*; Sheldrick, 1996 ▶) *T*_min_ = 0.740, *T*_max_ = 0.785
11121 measured reflections
4254 independent reflections
2027 reflections with *I* > 2σ(*I*)
*R*_int_ = 0.085

### Refinement  

*R*[*F* ^2^ > 2σ(*F* ^2^)] = 0.065
*wR*(*F* ^2^) = 0.149
*S* = 0.98
4254 reflections
244 parameters
H-atom parameters constrained
Δρ_max_ = 0.49 e Å^−3^
Δρ_min_ = −0.39 e Å^−3^

### 

Data collection: *APEX2* (Bruker, 2002 ▶); cell refinement: *SAINT* (Bruker, 2002 ▶); data reduction: *SAINT*; program(s) used to solve structure: *SHELXTL* (Sheldrick, 2008 ▶); program(s) used to refine structure: *SHELXTL*; molecular graphics: *Mercury* (Macrae *et al.*, 2006 ▶); software used to prepare material for publication: *SHELXTL* and *publCIF* (Westrip, 2010 ▶).

## Supplementary Material

Crystal structure: contains datablock(s) global, I. DOI: 10.1107/S1600536814015608/bt6986sup1.cif

Structure factors: contains datablock(s) I. DOI: 10.1107/S1600536814015608/bt6986Isup2.hkl

**Fig. 1.**
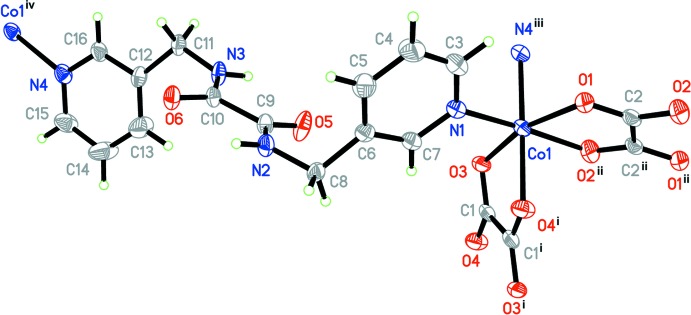
A view of the molecule of (I). Displacement ellipsoids are drawn at the 30% probability level. (i) - x + 1, -y, - z + 1; (ii) -x, -y, - z + 1; (iii) x, - y + 1/2, z + 1/2; (iv) x + 1, - y + 1/2, z - 1/2.

Click here for additional data file.x y z x y z x y z x y z . DOI: 10.1107/S1600536814015608/bt6986fig1.tif
A view of the mol­ecule of (I). Displacement ellipsoids are drawn at the 30% probability level. (i) − *x* + 1, −*y*, − *z* + 1; (ii) −*x*, −*y*, − *z* + 1; (iii) *x*, − *y* + 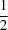, *z* + 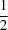; (iv) *x* + 1, − *y* + 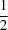, *z* − 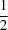.

**Fig. 2.**
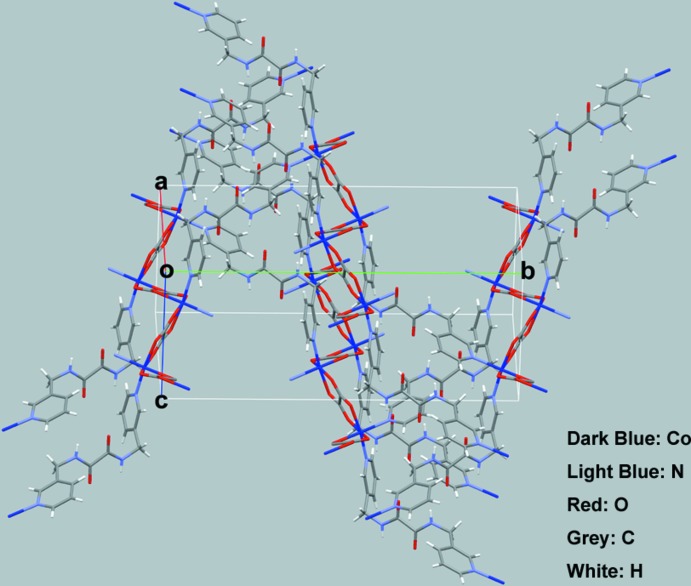
View of the three-dimensional structure of (I).

. DOI: 10.1107/S1600536814015608/bt6986fig2.tif
View of the three-dimensional structure of (I).

CCDC reference: 1012047

Additional supporting information:  crystallographic information; 3D view; checkCIF report

## Figures and Tables

**Table 1:** Hydrogen-bond geometry (Å, °)

*D*—H⋯*A*	*D*—H	H⋯*A*	*D*⋯*A*	*D*—H⋯*A*
N3—H3⋯O6^i^	0.86	2.14	2.863 (5)	142

Symmetry code: (i) 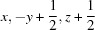.

## supplementary crystallographic information

### S1. Comment

Design of effective ligands and the proper choice of metal centers are the keys to design and construct novel metal-organic frameworks (Kitagawa *et al.*, 2004; Ma *et al.*, 2009). These complexes can be specially designed by the careful selection of metal cations with preferred coordination geometries, the nature of the anions, the structure of the connecting ligands, and the reaction conditions (Li *et al.*, 2005; Wang *et al.*, 2007). We selected oxalic acid as an organic carboxylate anion and *N*,*N*'-Bis-pyridin-4-ylmethyl-oxalamide (BPMO) as a N-donor neutral ligand, generating a coordination compound, [Co(C_2_O_4_)(BPMO)]_n_, which is reported here.

In the asymmetric unit of the title compound, [Co(C_2_O_4_)(BPMO)]_n_, the central Co^II^ is six-coordinated by two nitrogen atoms from different BPMO ligands and four oxygen atoms from two oxalate anions in a distorted octahedral coordination geometry. The Co—N and Co—O distances are comparable to those found in other crystallographically characterized Co^II^ complexes (Ma *et al.*, 2005). The Co^II^ atoms are linked by the oxalate anions to give a one-dimensional chain. The chains are linked by BPMO ligands and extend the chains into a three-dimensional supramolecular architecture. Moreover, the hydrogen bonds between the N-donor neutral ligand and oxalate, are crucial for stabilizing the three-dimensional framework.

### S2. Experimental

The synthesis was performed under hydrothermal conditions. A mixture of Co(CH_3_COO)_2_^.^4(H_2_O),(0.2 mmol, 0.05 g), *N*,*N*'-Bis-pyridin-4-ylmethyl-oxalamide (0.2 mmol, 0.054 g), sodium oxalate (0.2 mmol,0.026 g) and H_2_O(15 ml) in a 25 ml stainless steel reactor with a Teflon liner was heated from 293 to 443 K in 2 h and a constant temperature was maintained at 443 K for 72 h, after which the mixture was cooled to 298 K. Pink crystals of (I) were recovered from the reaction.

### S3. Refinement

All H atoms on C and N atoms atoms were poisitioned geometrically and refined as riding atoms with *U*_iso_(H) = 1.2 *U*_eq_(C, N).

### Crystal data

**Table:**

[Co(C_2_O_4_)(C_14_H_14_N_4_O_2_)]	*F*(000) = 852
*M**_r_* = 417.24	*D*_x_ = 1.581 Mg m^−^^3^
Monoclinic, *P*2_1_/*c*	Mo *K*α radiation, λ = 0.71073 Å
Hall symbol: -P 2ybc	Cell parameters from 4380 reflections
*a* = 8.4143 (12) Å	θ = 1.7–22.8°
*b* = 24.421 (4) Å	µ = 1.02 mm^−^^1^
*c* = 9.2884 (14) Å	*T* = 293 K
β = 113.322 (2)°	Block, pink
*V* = 1752.7 (4) Å^3^	0.43 × 0.25 × 0.25 mm
*Z* = 4	

### Data collection

**Table:**

Bruker SMART APEXII CCD diffractometer	4254 independent reflections
Radiation source: fine-focus sealed tube	2027 reflections with *I* > 2σ(*I*)
Graphite monochromator	*R*_int_ = 0.085
phi and ω scans	θ_max_ = 28.4°, θ_min_ = 2.5°
Absorption correction: multi-scan (*SADABS*; Sheldrick, 1996)	*h* = −11→10
*T*_min_ = 0.740, *T*_max_ = 0.785	*k* = −32→32
11121 measured reflections	*l* = −12→10

### Refinement

**Table:**

Refinement on *F*^2^	Primary atom site location: structure-invariant direct methods
Least-squares matrix: full	Secondary atom site location: difference Fourier map
*R*[*F*^2^ > 2σ(*F*^2^)] = 0.065	Hydrogen site location: inferred from neighbouring sites
*wR*(*F*^2^) = 0.149	H-atom parameters constrained
*S* = 0.98	*w* = 1/[σ^2^(*F*_o_^2^) + (0.0591*P*)^2^] where *P* = (*F*_o_^2^ + 2*F*_c_^2^)/3
4254 reflections	(Δ/σ)_max_ < 0.001
244 parameters	Δρ_max_ = 0.49 e Å^−^^3^
0 restraints	Δρ_min_ = −0.39 e Å^−^^3^

### Special details

**Table:**

Geometry. All e.s.d.'s (except the e.s.d. in the dihedral angle between two l.s. planes) are estimated using the full covariance matrix. The cell e.s.d.'s are taken into account individually in the estimation of e.s.d.'s in distances, angles and torsion angles; correlations between e.s.d.'s in cell parameters are only used when they are defined by crystal symmetry. An approximate (isotropic) treatment of cell e.s.d.'s is used for estimating e.s.d.'s involving l.s. planes.
Refinement. Refinement of *F*^2^ against ALL reflections. The weighted *R*-factor *wR* and goodness of fit *S* are based on *F*^2^, conventional *R*-factors *R* are based on *F*, with *F* set to zero for negative *F*^2^. The threshold expression of *F*^2^ > σ(*F*^2^) is used only for calculating *R*-factors(gt) *etc*. and is not relevant to the choice of reflections for refinement. *R*-factors based on *F*^2^ are statistically about twice as large as those based on *F*, and *R*- factors based on ALL data will be even larger.

### Fractional atomic coordinates and isotropic or equivalent isotropic displacement parameters (Å^2^)

**Table:**

	*x*	*y*	*z*	*U*_iso_*/*U*_eq_	
C1	0.5475 (6)	0.02677 (18)	0.5426 (6)	0.0391 (12)	
C2	−0.0727 (6)	0.00051 (18)	0.4182 (6)	0.0373 (11)	
C3	0.0325 (7)	0.0938 (2)	0.0435 (7)	0.0588 (15)	
H3A	−0.0628	0.0983	0.0692	0.071*	
C4	0.0141 (7)	0.1061 (2)	−0.1055 (7)	0.0644 (16)	
H4	−0.0906	0.1187	−0.1797	0.077*	
C5	0.1578 (7)	0.0990 (2)	−0.1424 (6)	0.0553 (15)	
H5	0.1507	0.1079	−0.2422	0.066*	
C6	0.3094 (6)	0.07904 (17)	−0.0329 (6)	0.0358 (11)	
C7	0.3141 (6)	0.06856 (18)	0.1129 (6)	0.0413 (12)	
H7	0.4172	0.0555	0.1884	0.050*	
C8	0.4646 (6)	0.06836 (16)	−0.0724 (6)	0.0398 (12)	
H8A	0.5578	0.0537	0.0195	0.048*	
H8B	0.4343	0.0409	−0.1543	0.048*	
C9	0.5876 (6)	0.15839 (19)	−0.0278 (6)	0.0372 (11)	
C10	0.6436 (5)	0.20856 (18)	−0.0958 (6)	0.0353 (11)	
C11	0.7359 (6)	0.30333 (18)	−0.0376 (6)	0.0411 (12)	
H11A	0.7224	0.3306	0.0328	0.049*	
H11B	0.6576	0.3130	−0.1432	0.049*	
C12	0.9189 (6)	0.30606 (19)	−0.0279 (5)	0.0396 (12)	
C13	1.0379 (7)	0.2660 (2)	0.0275 (7)	0.0700 (18)	
H13	1.0094	0.2334	0.0632	0.084*	
C14	1.2033 (7)	0.2734 (2)	0.0312 (8)	0.083 (2)	
H14	1.2859	0.2458	0.0666	0.100*	
C15	1.2411 (7)	0.3229 (2)	−0.0193 (7)	0.0631 (16)	
H15	1.3517	0.3281	−0.0165	0.076*	
C16	0.9683 (6)	0.35427 (19)	−0.0767 (6)	0.0431 (12)	
H16	0.8861	0.3819	−0.1153	0.052*	
N1	0.1806 (5)	0.07571 (16)	0.1551 (5)	0.0455 (11)	
N2	0.5257 (5)	0.11716 (15)	−0.1243 (4)	0.0403 (10)	
H2	0.5213	0.1190	−0.2183	0.048*	
N3	0.6855 (4)	0.25082 (15)	0.0010 (4)	0.0423 (10)	
H3	0.6826	0.2467	0.0918	0.051*	
N4	1.1276 (5)	0.36374 (16)	−0.0717 (5)	0.0439 (10)	
O1	−0.0491 (4)	0.02611 (12)	0.3106 (4)	0.0434 (8)	
O2	−0.2077 (4)	−0.02540 (13)	0.4033 (4)	0.0503 (9)	
O3	0.4618 (4)	0.07115 (12)	0.5055 (4)	0.0446 (9)	
O4	0.6991 (4)	0.02217 (12)	0.6401 (4)	0.0494 (9)	
O5	0.5999 (5)	0.15868 (13)	0.1070 (4)	0.0612 (11)	
O6	0.6455 (4)	0.20801 (12)	−0.2267 (4)	0.0464 (8)	
Co1	0.20072 (8)	0.05586 (2)	0.38091 (8)	0.0405 (2)	

### Atomic displacement parameters (Å^2^)

**Table:**

	*U*^11^	*U*^22^	*U*^33^	*U*^12^	*U*^13^	*U*^23^
C1	0.035 (3)	0.043 (3)	0.049 (3)	0.003 (2)	0.026 (3)	−0.001 (2)
C2	0.034 (3)	0.033 (2)	0.051 (3)	0.002 (2)	0.023 (2)	−0.008 (2)
C3	0.050 (4)	0.067 (4)	0.068 (4)	0.013 (3)	0.034 (3)	0.009 (3)
C4	0.043 (4)	0.087 (4)	0.053 (4)	0.018 (3)	0.009 (3)	0.015 (3)
C5	0.063 (4)	0.061 (4)	0.044 (4)	0.003 (3)	0.023 (3)	0.001 (3)
C6	0.037 (3)	0.026 (2)	0.043 (3)	−0.004 (2)	0.015 (3)	−0.005 (2)
C7	0.034 (3)	0.044 (3)	0.046 (3)	0.006 (2)	0.016 (2)	0.004 (3)
C8	0.053 (3)	0.027 (2)	0.048 (3)	−0.004 (2)	0.029 (3)	−0.001 (2)
C9	0.035 (3)	0.041 (3)	0.037 (3)	−0.010 (2)	0.016 (2)	−0.002 (2)
C10	0.034 (3)	0.039 (3)	0.038 (3)	−0.010 (2)	0.019 (2)	−0.001 (2)
C11	0.048 (3)	0.040 (3)	0.044 (3)	−0.010 (2)	0.027 (3)	0.001 (2)
C12	0.043 (3)	0.042 (3)	0.034 (3)	−0.013 (2)	0.016 (2)	−0.001 (2)
C13	0.052 (4)	0.045 (3)	0.109 (6)	−0.009 (3)	0.028 (4)	0.015 (3)
C14	0.050 (4)	0.046 (4)	0.142 (7)	0.003 (3)	0.026 (4)	0.016 (4)
C15	0.045 (3)	0.048 (3)	0.104 (5)	−0.003 (3)	0.036 (3)	−0.003 (3)
C16	0.039 (3)	0.045 (3)	0.052 (3)	−0.006 (2)	0.024 (3)	0.001 (3)
N1	0.041 (3)	0.047 (2)	0.054 (3)	0.0103 (19)	0.024 (2)	0.009 (2)
N2	0.052 (3)	0.041 (2)	0.035 (2)	−0.0080 (19)	0.026 (2)	−0.0039 (19)
N3	0.052 (3)	0.045 (2)	0.038 (3)	−0.0204 (19)	0.026 (2)	−0.005 (2)
N4	0.039 (2)	0.042 (2)	0.058 (3)	−0.0088 (19)	0.027 (2)	−0.005 (2)
O1	0.040 (2)	0.044 (2)	0.048 (2)	0.0012 (15)	0.0202 (17)	0.0061 (17)
O2	0.042 (2)	0.057 (2)	0.055 (2)	−0.0090 (17)	0.0222 (18)	−0.0050 (18)
O3	0.0356 (19)	0.0339 (18)	0.070 (3)	0.0063 (14)	0.0269 (18)	0.0041 (16)
O4	0.037 (2)	0.041 (2)	0.065 (3)	0.0040 (16)	0.0145 (19)	−0.0052 (17)
O5	0.098 (3)	0.050 (2)	0.044 (2)	−0.036 (2)	0.037 (2)	−0.0086 (18)
O6	0.064 (2)	0.044 (2)	0.041 (2)	−0.0107 (16)	0.0318 (19)	−0.0026 (16)
Co1	0.0349 (4)	0.0385 (4)	0.0551 (5)	0.0057 (3)	0.0253 (3)	0.0030 (3)

### Geometric parameters (Å, º)

**Table:**

C1—O4	1.243 (5)	C11—N3	1.440 (5)
C1—O3	1.271 (5)	C11—C12	1.508 (6)
C1—C1^i^	1.573 (9)	C11—H11A	0.9700
C2—O2	1.259 (5)	C11—H11B	0.9700
C2—O1	1.260 (5)	C12—C13	1.348 (6)
C2—C2^ii^	1.527 (9)	C12—C16	1.383 (6)
C3—N1	1.342 (6)	C13—C14	1.390 (7)
C3—C4	1.363 (7)	C13—H13	0.9300
C3—H3A	0.9300	C14—C15	1.380 (7)
C4—C5	1.392 (7)	C14—H14	0.9300
C4—H4	0.9300	C15—N4	1.332 (6)
C5—C6	1.369 (6)	C15—H15	0.9300
C5—H5	0.9300	C16—N4	1.343 (5)
C6—C7	1.364 (6)	C16—H16	0.9300
C6—C8	1.512 (6)	N1—Co1	2.093 (4)
C7—N1	1.339 (5)	N2—H2	0.8600
C7—H7	0.9300	N3—H3	0.8600
C8—N2	1.454 (5)	N4—Co1^iii^	2.155 (4)
C8—H8A	0.9700	O1—Co1	2.070 (3)
C8—H8B	0.9700	O2—Co1^ii^	2.117 (3)
C9—O5	1.215 (5)	O3—Co1	2.072 (3)
C9—N2	1.311 (5)	O4—Co1^i^	2.124 (3)
C9—C10	1.535 (6)	Co1—O2^ii^	2.117 (3)
C10—O6	1.222 (5)	Co1—O4^i^	2.124 (3)
C10—N3	1.322 (5)	Co1—N4^iv^	2.155 (4)
			
O4—C1—O3	125.7 (4)	C12—C13—H13	120.0
O4—C1—C1^i^	117.5 (5)	C14—C13—H13	120.0
O3—C1—C1^i^	116.8 (6)	C15—C14—C13	117.9 (5)
O2—C2—O1	125.5 (4)	C15—C14—H14	121.0
O2—C2—C2^ii^	115.7 (6)	C13—C14—H14	121.0
O1—C2—C2^ii^	118.8 (5)	N4—C15—C14	123.5 (5)
N1—C3—C4	123.7 (5)	N4—C15—H15	118.2
N1—C3—H3A	118.2	C14—C15—H15	118.2
C4—C3—H3A	118.2	N4—C16—C12	124.1 (4)
C3—C4—C5	117.5 (5)	N4—C16—H16	117.9
C3—C4—H4	121.2	C12—C16—H16	117.9
C5—C4—H4	121.2	C7—N1—C3	116.5 (5)
C6—C5—C4	120.4 (5)	C7—N1—Co1	121.2 (3)
C6—C5—H5	119.8	C3—N1—Co1	122.2 (3)
C4—C5—H5	119.8	C9—N2—C8	120.0 (4)
C7—C6—C5	117.2 (4)	C9—N2—H2	120.0
C7—C6—C8	121.4 (4)	C8—N2—H2	120.0
C5—C6—C8	121.5 (4)	C10—N3—C11	123.5 (4)
N1—C7—C6	124.7 (5)	C10—N3—H3	118.3
N1—C7—H7	117.7	C11—N3—H3	118.3
C6—C7—H7	117.7	C15—N4—C16	116.4 (4)
N2—C8—C6	113.1 (3)	C15—N4—Co1^iii^	122.3 (3)
N2—C8—H8A	109.0	C16—N4—Co1^iii^	120.8 (3)
C6—C8—H8A	109.0	C2—O1—Co1	112.5 (3)
N2—C8—H8B	109.0	C2—O2—Co1^ii^	112.7 (3)
C6—C8—H8B	109.0	C1—O3—Co1	111.1 (3)
H8A—C8—H8B	107.8	C1—O4—Co1^i^	110.2 (3)
O5—C9—N2	123.9 (4)	O1—Co1—O3	163.58 (13)
O5—C9—C10	120.3 (4)	O1—Co1—N1	95.51 (15)
N2—C9—C10	115.8 (4)	O3—Co1—N1	99.50 (14)
O6—C10—N3	125.5 (4)	O1—Co1—O2^ii^	79.59 (13)
O6—C10—C9	121.8 (4)	O3—Co1—O2^ii^	84.77 (12)
N3—C10—C9	112.7 (4)	N1—Co1—O2^ii^	172.33 (14)
N3—C11—C12	114.8 (4)	O1—Co1—O4^i^	92.68 (12)
N3—C11—H11A	108.6	O3—Co1—O4^i^	80.86 (12)
C12—C11—H11A	108.6	N1—Co1—O4^i^	89.62 (14)
N3—C11—H11B	108.6	O2^ii^—Co1—O4^i^	84.76 (13)
C12—C11—H11B	108.6	O1—Co1—N4^iv^	92.74 (13)
H11A—C11—H11B	107.6	O3—Co1—N4^iv^	92.70 (13)
C13—C12—C16	117.9 (4)	N1—Co1—N4^iv^	94.43 (15)
C13—C12—C11	125.3 (4)	O2^ii^—Co1—N4^iv^	91.71 (14)
C16—C12—C11	116.8 (4)	O4^i^—Co1—N4^iv^	172.90 (15)
C12—C13—C14	120.0 (5)		
			
N1—C3—C4—C5	0.2 (9)	C14—C15—N4—Co1^iii^	171.3 (5)
C3—C4—C5—C6	1.7 (8)	C12—C16—N4—C15	1.5 (8)
C4—C5—C6—C7	−2.3 (7)	C12—C16—N4—Co1^iii^	−171.1 (4)
C4—C5—C6—C8	177.0 (5)	O2—C2—O1—Co1	−173.9 (3)
C5—C6—C7—N1	1.0 (7)	C2^ii^—C2—O1—Co1	5.5 (6)
C8—C6—C7—N1	−178.3 (4)	O1—C2—O2—Co1^ii^	−174.5 (3)
C7—C6—C8—N2	−121.0 (5)	C2^ii^—C2—O2—Co1^ii^	6.2 (6)
C5—C6—C8—N2	59.8 (6)	O4—C1—O3—Co1	−166.2 (4)
O5—C9—C10—O6	174.2 (4)	C1^i^—C1—O3—Co1	13.8 (6)
N2—C9—C10—O6	−6.6 (6)	O3—C1—O4—Co1^i^	−166.7 (4)
O5—C9—C10—N3	−6.6 (6)	C1^i^—C1—O4—Co1^i^	13.4 (6)
N2—C9—C10—N3	172.6 (4)	C2—O1—Co1—O3	11.5 (6)
N3—C11—C12—C13	−6.9 (7)	C2—O1—Co1—N1	167.5 (3)
N3—C11—C12—C16	174.7 (4)	C2—O1—Co1—O2^ii^	−6.5 (3)
C16—C12—C13—C14	−1.3 (9)	C2—O1—Co1—O4^i^	77.7 (3)
C11—C12—C13—C14	−179.7 (5)	C2—O1—Co1—N4^iv^	−97.7 (3)
C12—C13—C14—C15	1.6 (10)	C1—O3—Co1—O1	51.8 (6)
C13—C14—C15—N4	−0.3 (10)	C1—O3—Co1—N1	−104.0 (3)
C13—C12—C16—N4	−0.3 (8)	C1—O3—Co1—O2^ii^	69.6 (3)
C11—C12—C16—N4	178.2 (4)	C1—O3—Co1—O4^i^	−15.9 (3)
C6—C7—N1—C3	0.9 (7)	C1—O3—Co1—N4^iv^	161.1 (3)
C6—C7—N1—Co1	178.1 (3)	C7—N1—Co1—O1	−142.6 (3)
C4—C3—N1—C7	−1.5 (8)	C3—N1—Co1—O1	34.4 (4)
C4—C3—N1—Co1	−178.7 (4)	C7—N1—Co1—O3	30.7 (4)
O5—C9—N2—C8	0.8 (7)	C3—N1—Co1—O3	−152.3 (4)
C10—C9—N2—C8	−178.3 (4)	C7—N1—Co1—O2^ii^	−92.7 (11)
C6—C8—N2—C9	64.2 (6)	C3—N1—Co1—O2^ii^	84.3 (11)
O6—C10—N3—C11	1.6 (7)	C7—N1—Co1—O4^i^	−50.0 (4)
C9—C10—N3—C11	−177.5 (4)	C3—N1—Co1—O4^i^	127.0 (4)
C12—C11—N3—C10	−77.4 (6)	C7—N1—Co1—N4^iv^	124.2 (4)
C14—C15—N4—C16	−1.2 (9)	C3—N1—Co1—N4^iv^	−58.8 (4)

Symmetry codes: (i) −*x*+1, −*y*, −*z*+1; (ii) −*x*, −*y*, −*z*+1; (iii) *x*+1, −*y*+1/2, *z*−1/2; (iv) *x*−1, −*y*+1/2, *z*+1/2.

### Hydrogen-bond geometry (Å, º)

**Table:**

*D*—H···*A*	*D*—H	H···*A*	*D*···*A*	*D*—H···*A*
N3—H3···O6^v^	0.86	2.14	2.863 (5)	142

Symmetry code: (v) *x*, −*y*+1/2, *z*+1/2.
