# Supplementary figures and images for: Crystal structure of poly[{μ-N,N′-bis[(pyridin-4-yl)meth­yl]oxalamide}-μ-oxalato-cobalt(II)]
Source: Acta Crystallogr Sect E Struct Rep Online. 2014 Aug 1;70(Pt 9):m307–8. doi: 10.1107/S1600536814015608 (PMC4186178; doi:10.1107/S1600536814015608)

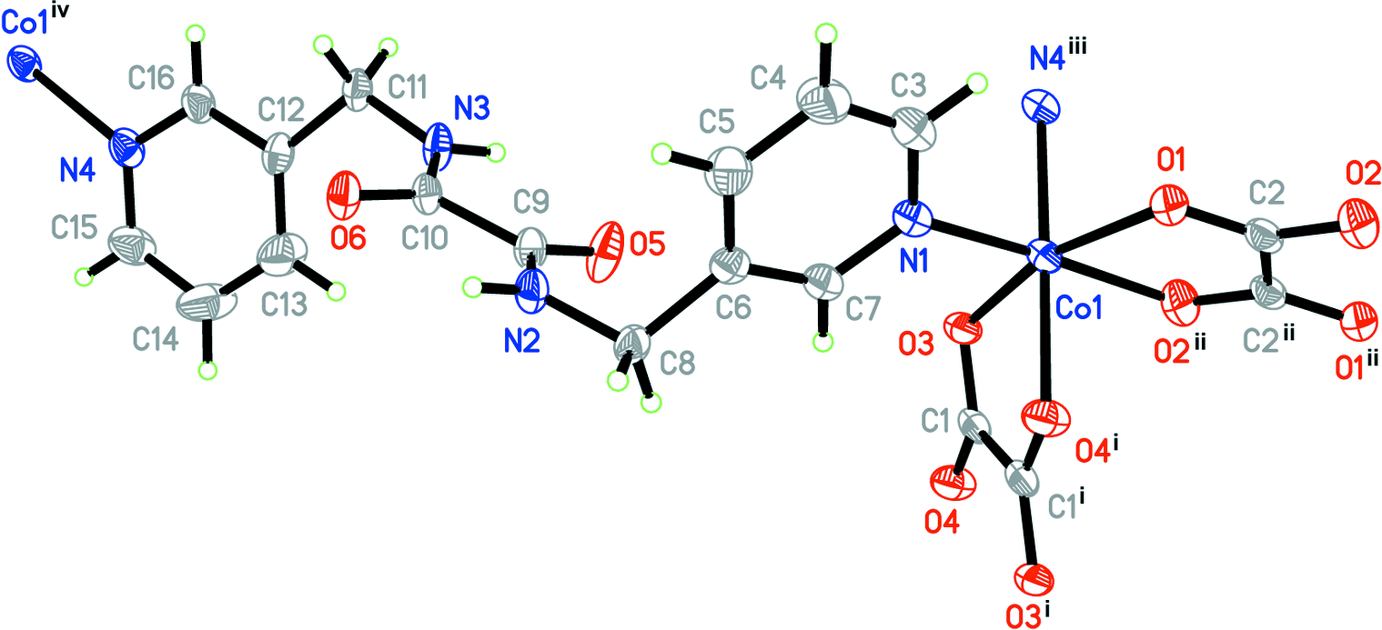

Supplement: Supplementary file 3 [file e-70-0m307-fig1.tif]

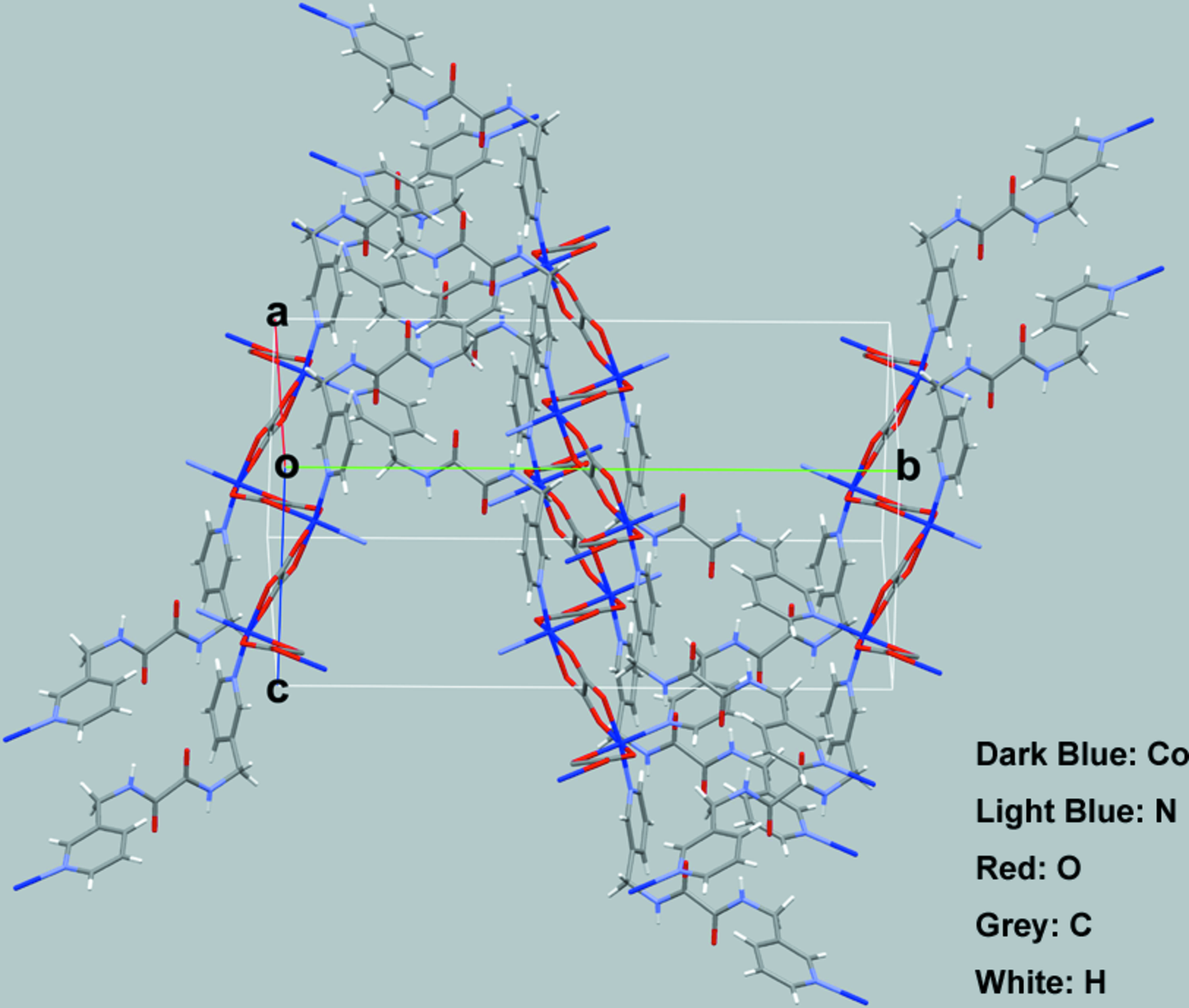

Supplement: Supplementary file 4 [file e-70-0m307-fig2.tif]
